# Design, Synthesis and Biological Evaluation of *N*-Sulfonyl Homoserine Lactone Derivatives as Inhibitors of Quorum Sensing in *Chromobacterium violaceum*

**DOI:** 10.3390/molecules18033266

**Published:** 2013-03-13

**Authors:** Mingming Zhao, Yingying Yu, Yuhui Hua, Fan Feng, Yigang Tong, Xiaohong Yang, Junhai Xiao, Hongrui Song

**Affiliations:** 1 School of Pharmaceutical Engineering, Shenyang Pharmaceutical University, 103 Wenhua Road, Shenhe District, Shenyang 110016, China; E-Mail: zhaomingmingbest@163.com; 2 Laboratory of Computer-Aided Drug Design & Discovery, Beijing Institute of Pharmacology & Toxicology, Beijing 100850, China; E-Mails: yuyingying10@163.com (Y.Y.); fengfanbio@126.com (F.F.); 3 School of Pharmacutical Sciences, Jilin University, Changchun 130021, China; E-Mail; xiaohongyang88@126.com; 4 State Key Laboratory of Pathogen and Biosecurity, Beijing Institute of Microbiology and Epidemiology, Beijing 100071, China; E-Mails: yuhui_hua@126.com (Y.H.); tong.yigang@gmail.com (Y.T.)

**Keywords:** *N*-sulfonyl homoserine lactone, quorum sensing inhibitor, design, synthesis

## Abstract

A novel series of *N*-sulfonyl homoserine lactone derivatives **5a**–**l** has been designed, synthesized and evaluated for quorum sensing inhibitory activities towards violacein production. Of the compounds synthesized, compound **5h** was found to possess an excellent level of enantiopurity (99.2% e.e.). The results indicated that compounds bearing an *ortho* substituent on their phenyl ring exhibited excellent levels of inhibitory activity against violacein production. Compounds **5h** and **5k** in particular, with IC_50_ values of 1.64 and 1.66 µM, respectively, were identified as promising lead compounds for further structural modification.

## 1. Introduction

Quorum sensing (QS) is a cell communication mechanism involving the production, release and detection of small signaling molecules called autoinducers (AIs). AIs can activate specific receptors associated with transcription signals that are responsible for controlling a variety of different biochemical processes [1,2,3]. Several important phenotypes are effectively regulated by QS, including bioluminescence, virulence expression, and biofilm formation [4,5,6].

Recent increases in the number of bacterial strains resistant to antibiotics have emphasized the need for the development of a new generation of antibacterial agents. QS inhibitors can provide insights into bacterial signaling processes from both the fundamental and applied perspectives, allowing for the discovery of new strategies for the development of novel antibacterial agents [7,8,9,10,11].

**Figure 1:**
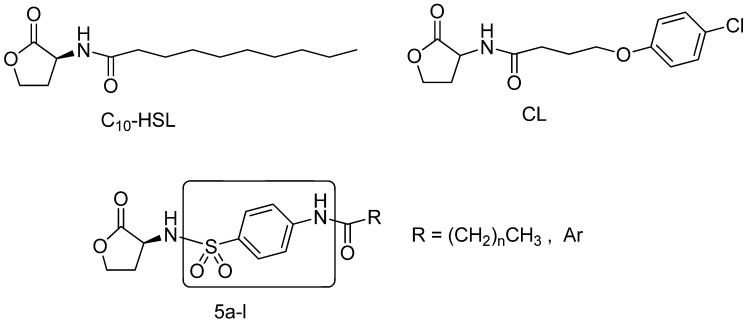
Structures of C_10_-HSL, CL and target compounds **5a**–**l**.

*Chromobacterium violaceum* is a soil borne Gram-negative bacteria that synthesizes the violet pigment violacein as a result of QS using its AI *N*-hexanoyl homoserine lactone (C_6_-HSL) [12]. *C.* *violaceum* CV026 (CV026) has been widely applied as a model bacterial to screen new QS inhibitors [13]. A significant number of novel *N*-acyl homoserine lactone derivatives have been developed in succession on the basis of their inhibition of violacein production. C_10_-HSL and Chloro Lactone (CL) are currently the most effective compounds to inhibit violacein production reported to date [14,15] (Figure 1). These compounds can readily access cell membranes because they possess a hydrophilic homoserine lactone ring together with a hydrophobic alky or aryl group [16]. The mechanism of inhibiting violacein production was that C_10_-HSL allowed DNA binding but reduced or eliminated transcriptional activation, suggesting that the CviR-C_10_-HSL complex could not productively interact with RNA polymerase. But CL prevented CviR from binding DNA [14].

Among a great number of *N*-acyl homoserine lactones QS inhibitors, only few studies have focused on developing QS inhibitors via the amide moiety [17]. To investigate the function of the amide groups, this group was replaced by heterocyclic rings, sulfonamides and reverse-amides [9,18,19,20]. The amide group was replaced by triazoles and tetrazoles, because they were nonclassical bioisosteres and displayed some similarity with amide bonds [19,20]. Castang *et al*. synthesized a series of *N*-sulfonyl-homoserine lactones which exhibit QS inhibitory activity in *Vibrio fischeri* because of the widespread biological activity of sulfonamides [18]. The reverse-amide was a new idea for QS inhibitor research [9]. It has been reported that acyl tail lengths are important factors in determining the QS inhibitory activities of the compounds [13,19]. Generally, *N*-acyl homoserine lactone analogues which have a long acyl tail chain or aryl group in the tail chain were found to possess QS inhibitory activity [13,14,18]. Based on above reports, in order to improve the QS inhibitory activity of a lead compound, the amide group should be replaced by similar bioisosteres, and the compound should have a long acyl tail chain or aryl group in tail chain.

To investigate the activities of novel QS inhibitors towards violacein production, we have made several changes to the structures of the known inhibitors C_10_-HSL and CL, whilst retaining the key homoserine lactone. These changes included the introduction of a 4-aminobenzenesulfonyl moiety between the nucleus and the acyl side chain. The decision to introduce a sulfonyl moiety was based on the fact that sulfonamides generally exhibit a broad range of biological activities [18] and are also a bioisostere of the amide group. It was also envisaged that the introduction of a benzene ring moiety could lead to an increase in the hydrophobicity of the compounds. To examine the effects of the alkyl and aryl groups on the QS inhibitory activities of the compounds following the introduction of the 4-aminobenzenesulfonyl moiety, a series of novel *N*-sulfonyl homoserine lactone derivatives **5a**–**l** was designed, synthesized and valuated as potential inhibitors QS according to their ability to inhibit violacein production, and a preliminary structure-activity relationship (SAR) study of these compounds was conducted.

## 2. Results and Discussion

### 2.1. Chemistry

**Scheme 1:**
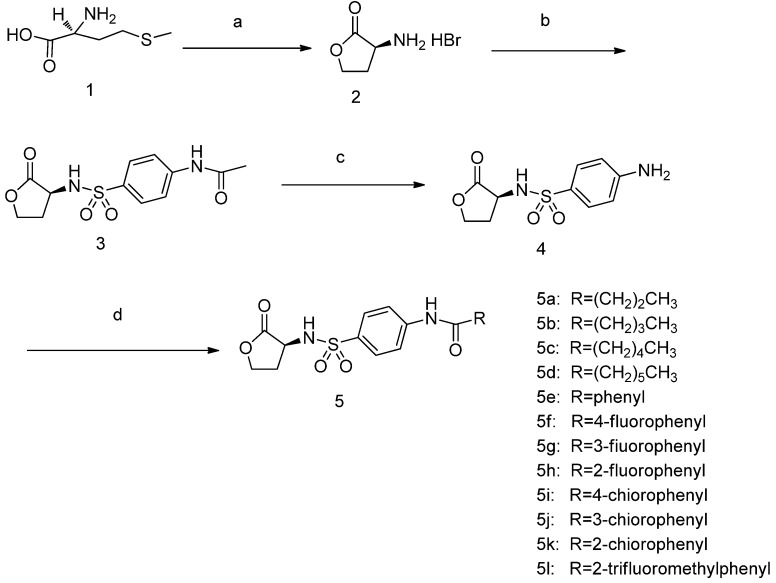
Synthesis of target compounds.

All the agents, unless mentioned otherwise, were commercially available and were directly used without further purification. The syntheses of the intermediates and target compounds were accomplished according to the steps illustrated in Scheme 1. Commercially available L-methionine (**1**) was reacted with bromoacetic acid at reflux to give compound **2**, which was subsequently reacted with 4-acetamidobenzenesulfonyl chloride in the presence of catalytic triethylamine (TEA) to give compound **3**. Treatment of compound **3** with refluxing in 6 N HCl for 3 h produced the hydrolysis product compound **4**, which was reacted with a series of commercially available acid chlorides to afford the desired target compounds **5a**–**l**.

It is noteworthy that compound **5h** was synthesized according to this route with an excellent level of enantiomeric purity (*i.e.*, an enantiomeric excess of 99.2% as determined by chiral HPLC), demonstrating the utility of the current method for the production of compounds with high enantiomeric purities. Unfortunately however, the enantiomeric purities of *N*-sulfonyl homoserine lactone derivatives synthesized according to other known procedures are not always provided, precluding a comparison between the different methods from this perspective.

*Reagents and Conditions*: (a) bromoacetic acid, acetic acid, H_2_O, 2-propanol, reflux, then concentrated HCl, 50 °C, 10 min; (b) 4-acetamidobenzenesulfonyl chloride, TEA, EtOH, 0 °C to r.t., overnight; (c) 6 N HCl, EtOH, reflux, 3 h; (d) ClCOR, CH_2_Cl_2_, 0 °C to r.t. overnight.

### 2.2. Biological Results and Discussion

#### 2.2.1. Antibacterial Activity

**Figure 2:**
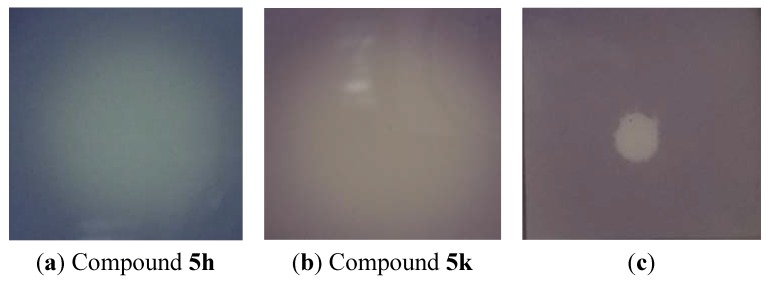
Antibacterial spot tests of compounds **5h**,**k**.

The spot test method was performed to test antibacterial activity of compound **5a**–**l** against *C. violaceum* CV026. This is important as QS inhibition is focused on the interference of bacterial signaling and not on antibacterial activity. The results of the spot test showed the white colonies/holes in the purple background of the plates and no growth inhibition zone in the highest concentration of compound **5h**,**k** (Figure 2a,b), and the same phenomenon had been observed for other compounds. In comparison with them, Figure 2c showed the growth inhibition zone. This indicated that compound **5a**–**l** has no effect on the growth of *C. violaceum* CV026.

The concentrations of compounds **5h**,**k** were 0.0662M and 0.0647M respectively. The violacein was produced by C6-HSL and neither of compounds **5h**,**k** showed growth inhibition zones.

#### 2.2.2. Quorum Sensing Inhibition

The QS inhibitory activities of the target compounds **5a**–**l** were evaluated with CV026 based on their inhibition of violacein production, with compound C_10_-HSL being used as a positive control. The IC_50_ values for each of the synthesized compound have been summarized in Table 1.

**Table 1:** The substituents and IC_50_ values of **5a**–**l** for inhibiting violacein production.

Compound	R	IC_50_(μM)
**5a**	(CH_2_)_2_CH_3_	NA ^a^
**5b**	(CH_2_)_3_CH_3_	48.49 ± 2.68
**5c**	(CH_2_)_4_CH_3_	29.00 ± 2.41
**5d**	(CH_2_)_5_CH_3_	NA ^a^
**5e**	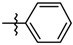	17.25 ± 2.60
**5f**	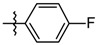	14.72 ± 2.58
**5g**	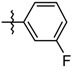	7.79 ± 2.68
**5h**	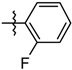	1.64 ± 0.27
**5i**	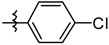	15.86 ± 2.35
**5j**	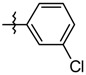	8.56 ± 1.25
**5k**	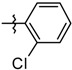	1.66 ± 0.33
**5l**	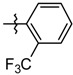	4.91 ± 0.97
**C_10_-HSL**		0.32 ± 0.07

^a^ NA means no significant inhibition.

As shown in Table 1, all of the newly synthesized compounds except **5a** and **5d** exhibited considerable levels of inhibitory activity against violacein production, with IC_50_ values ranging from 1.64–48.49 μM. Of these compounds, compounds **5h** and **5k** exhibited the greatest inhibitory activities with IC_50_ values of 1.64 and 1.66 μM, respectively, which highlighted the potential of these compounds as lead structures for further research towards the development of novel QS inhibitors.

Consideration of the SAR derived from the synthesized compounds suggested that compounds **5b**, **5c** and **5e**–**l**, which were designed based on the introduction of the 4-aminobenzenesulfonyl moiety between the homoserine lactone and acyl side chain of C_6_-HSL and CL, all exhibited considerable levels of inhibitory activity against violacein production. When the group at the R position was an alkyl group, the activity was dependent on the length of the chain, with a chain length of five carbon atoms such as **5c** providing the highest level of inhibitory activity amongst all these compounds. The introduction of an aryl group at the R position provided enhanced levels of inhibitory activity relative to the alkyl groups, as exemplified by the comparison of compound **5e** with **5c**. Further analysis of the compounds substituted at the R position with aryl groups revealed that *ortho* substituted aryl groups bearing electron withdrawing groups were superior to the corresponding *para* and *meta* substituted aryl groups. Compounds **5h** and **5k** containing *ortho*-halogen substituted aryl groups at the R position showed the highest levels of inhibitory activity of all of the analogues.

#### 2.2.3. Molecular Docking Studies

**Figure 3:**
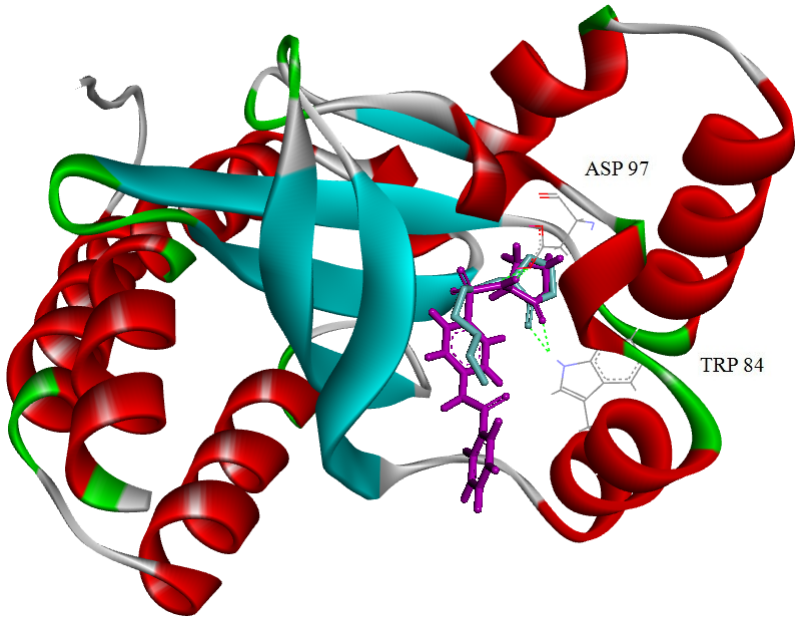
Molecular model of CviR with C_6_-HSL and compound **5h** bound to the active site.

A molecular docking study was conducted with the CviR structure (PDB entry 3QP1 and 3QP4), with C_6_-HSL and compound **5h** being docked into the structure using DOCK 4.0 [15]. The AI C_6_-HSL could bind to the CviR from *C.* *violaceum* to effectively regulate violacein production. As shown in Figure 3, compound **5h** occupied the space to that of the natural ligand C_6_-HSL. Both of them bound to CviR through hydrogen bonding interactions with the Trp 84 and Asp 97 residues.

C_6_-HSL and compound **5h** have been shown as cyan and purple stick models, respectively. The key amino acid residues providing binding interactions with C_6_-HSL and compound **5h** have been depicted as their respective chemical structures. Green dotted lines have been used to represent the hydrogen bonding interactions between the compounds and the amino acid residues (Trp 84 and Asp 97).

## 3. Experimental

### 3.1. General

Melting points were determined using a YRT-3 melting point detector (P.I.F. Tianjin University, Tianjin, China) and have been reported as uncorrected values. Optical rotation measurements were obtained on an Polaar 3005 polarimeter (Optical Activity Ltd., Cambridgeshire, UK) using a 10 cm path length micro cell. Chiral HPLC analysis was performed on a Shimadzu LC20 apparatus (Shimadzu, Kyoto, Japan). The ^1^H-NMR (400 MHz) and ^13^C-NMR (100 MHz) spectra were recorded using a Bruker ARX 400 spectrometer (Karlsruhe, Germany). The mass spectra were determined using an Agilent 5875 (EI) spectrometer (Palo Alto, CA, USA). All solvents and reagents were purchased commercially and used without further purification.

### 3.2. Chemical Synthesis

#### 3.2.1. Synthesis of Compounds **2**–**5**

*(S)-3-Aminodihydrofuran-2(3H)-one hydrobromide* (**2**). Compound **2** was synthesized according to a well-established literature procedure [21]. White solid; Yield 75%; m.p. 220–224 °C, (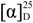 = −24.5° (*c* 0.10, H_2_O); lit. ref. [21] = −25.3° (*c* 0.087, H_2_O); ^1^H-NMR (DMSO-*d_6_*): δ 8.76 (3H, s), 4.45 (1H, t, *J* = 8.0 Hz, *J* = 7.6 Hz), 4.34–4.31 (2H, m), 2.55–2.51 (1H, m), 2.30–2.28 (1H, m); EI-MS: *m/z* = 102.1 [M+H]^+^.

*(S)-N-(4-(N-(2-Oxotetrahydrofuran-3-yl)sulfamoyl)phenyl)acetamide* (**3**). *N*-Acetylsulfanilyl chloride (12.3 g, 52 mmol) was added to a stirred suspension of triethylamine (8.7 g, 86 mmol) and compound **2** (8 g, 43 mmol) in ethanol (80 mL) at 0 °C in a portion-wise manner. The resulting mixture was stirred overnight at ambient temperature and then poured into ice-cold water (200 mL) with vigorous stirring. After stirring for 1 h, the white solid precipitate was collected by filtration. The filter cake was washed with ice-cold water before being dried under vacuum and recrystallized from ethanol to give the desired product **3**. (7.47g, 57%); m.p.174–176 °C; ^1^H-NMR (DMSO-*d_6_*): δ 10,33 (1H, s), 8.16 (1H, *J* = 8.0 Hz), 7.75 (4H, s), 4.34–4.31 (1H, m), 4.21–4.20 (1H, m), 4.08–4.07 (1H, m), 2.08–2.06 (4H, m), 1.82–1.78 (1H, m); EI-MS: *m/z* = 299.34 [M+H]^+^.

*(S)-4-Amino-N-(2-oxotetrahydrofuran-3-yl)benzenesulfonamide* (**4**). An aqueous 6 N solution of HCl (12 mL) was cautiously added with stirring to a solution of compound **3** (6.0 g, 20.1 mmol) in ethanol (25 mL). After heating under reflux for 3 h, the reaction mixture was then cooled and evaporated to dryness *in vacuo* to give the crude product as a residue, which was dissolved in water (100 mL). The pH of the solution was adjusted to 8–9 using a 1 N aqueous NH_4_OH solution, and the resulting mixture was stirred for 1 h resulting in the precipitation of a white solid. The white solid was collected by filtration and the filter cake washed with ice-cold water before being dried under vacuum and recrystallized from ethanol to give desired product **4** (3.4 g, 63%); m.p. 163–165 °C; ^1^H-NMR (DMSO-*d_6_*): δ 7.72 (1H, d, *J* = 9.2 Hz), 7.45 (2H, d, *J* = 8.4 Hz), 6.61 (2H, d, *J* = 8.8 Hz), 5.95 (2H, s), 4.22–4.18 (2H, m), 4.11–4.08 (1H, m), 2.07–2.04 (1H, m), 1.80–1.76 (1H, m); EI-MS: *m/z* = 256.4 [M+H]^+^.

#### 3.2.2. General Procedure for of the Preparation of Compounds **5**–**10**

*(S)-N-(4-(N-(2-Oxotetrahydrofuran-3-yl)sulfamoyl)phenyl)butyramide* (**5a**). Butyryl chloride (0.24 g, 2.3 mmol) was added to a solution of triethylamine (0.38 g, 3.8 mmol) and compound **4** (0.5 g, 1.9 mmol) in dry CH_2_Cl_2_ (10 mL) at 0 °C in a portion-wise manner, and the resulting mixture was stirred at ambient temperature overnight. The solvent was then removed under reduced pressure to give the crude product as a residue, which was purified by column chromatography (MeOH/DCM v/v = 1:40 silica) to provide the desired product **5a** (0.31 g, 51.0%); m.p. 180–182 °C; 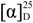 = +4.5° (c = 0.10, CH_3_OH); ^1^H-NMR (DMSO-d_6_): δ 10.28 (1H, s), 8.17 (1H, d, *J* = 8.0 Hz), 7.77–7.74 (4H, m), 4.34–4.32 (1H, m), 4.21–4.19 (1H, m), 4.10–4.07 (1H, m), 2.35–2.32 (2H, m), 2.11–2.08 (1H, m), 1.81–1.79 (1H, m), 1.64–1.58 (2H, m), 0.91(3H, t, *J* = 7.3 Hz, *J* = 7.6 Hz); ^1^^3^C-NMR (DMSO-*d_6_*): δ 174.5, 171.9, 142.9, 134.9, 127.7, 118.6, 65.1, 51.3, 38.4, 30.8, 29.4, 18.4, 13.6; EI-MS: *m/z* = 327.4 [M+H]^+^; ESI-HRMS: *m/z* [M+H]^+^ calcd for C_14_H_19_N_2_O_5_S: 327.1015; Found: 327.1013.

*(S)-N-(4-(N-(2-Oxotetrahydrofuran-3-yl)sulfamoyl)phenyl)pentanamide* (**5b**). Compound **5b** was obtained as a white solid (45.4% yield) from compound **4** according to the same procedure used for the synthesis of **5a**. m.p. 174–176 °C; 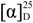 = +5.7° (c = 0.08, CH_3_OH); ^1^H-NMR (DMSO-*d_6_*): δ 10.28 (1H, s), 8.17 (1H, d, *J* = 9.6 Hz), 7.76–7.73 (4H, m), 4.34–4.33 (1H, m), 4.21–4.19 (1H, m), 4.10–4.07 (1H, m), 2.35–2.30 (2H, m), 2.09–2.08 (1H, m), 1.81–1.79 (1H, m), 1.59–1.55 (1H, m), 1.33–1.31 (1H, m), 0.89 (3H, t, *J* = 8.0 Hz, *J* = 8.0 Hz); ^1^^3^C-NMR (DMSO-*d_6_*): δ 174.5, 172.2, 143.0, 134.9, 127.7, 118.6, 65.1, 51.3, 36.2, 30.8, 29.4, 27.1, 21.8, 13.8; EI-MS: *m/z* = 341.4 [M+H]^+^; ESI-HRMS: *m/z* [M+H]^+^ calcd for C_15_H_21_N_2_O_5_S: 341.1171; Found: 341.1176.

*(S)-N-(4-(N-(2-Oxotetrahydrofuran-3-yl)sulfamoyl)phenyl)hexanamide* (**5c**). Compound **5c** was obtained as a white solid (48.3% yield) from compound **4** according to the same procedure used for the synthesis of **5a**. m.p. 142–143 °C; 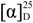 = +6.4° (c = 0.10, CH_3_OH); ^1^H-NMR (DMSO-*d_6_*): δ 10.24 (1H, s), 8.13 (1H, d, *J* = 9.2 Hz), 7.76–7.73 (4H, m), 4.34–4.31 (1H, m), 4.21–4.19 (1H, m), 4.10–4.09 (1H, m), 2.36–2.32 (2H, m), 2.11–2.09 (1H, m), 1.81–1.79 (1H, m) 1.61–1.57 (2H, m), 1.30–1.29 (4H, m), 0.89–0.86 (3H, m); ^1^^3^C-NMR (DMSO-d_6_): δ 174.5, 171.9, 142.9, 134.9, 127.6, 118.5, 65.1, 51.3, 36.4, 30.8, 29.4, 24.6, 21.9; EI-MS: *m/z* 355.4 [M+H]^+^; ESI-HRMS: *m/z* [M+H]^+^ calcd for C_16_H_23_N_2_O_5_S: 355.1328; Found: 355.1326.

*(S)-N-(4-(N-(2-Oxotetrahydrofuran-3-yl)sulfamoyl)phenyl)heptanamide* (**5d**). Compound **5d** was obtained as a white solid (45.4% yield) from compound **4** according to the same procedure used for the synthesis of **5a**. m.p. 143–144 °C; 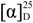 = +4.7° (c = 0.10, CH_3_OH); ^1^H-NMR (DMSO-*d_6_*): δ 10.26 (1H, s), 8.16 (1H, d, *J* = 9.2 Hz), 7.76–7.73 (4H, m), 4.34–4.31 (1H, m), 4.22–4.21 (1H, m), 4.11–4.07 (1H, m), 2.35–2.32 (2H, m), 2.11–2.09 (1H, m), 1.81–1.79 (1H, m), 1.60–1.57 (2H, m), 1.32–1.27 (6H, m), 0.88–0.84 (3H, m); ^1^^3^C-NMR (DMSO-d_6_): δ 174.5, 171.9, 142.9, 134.9, 127.7, 118.5, 65.1, 51.3, 36.5, 31.0, 29.4, 28.3, 24.9, 21.9, 13.9; EI-MS: *m/z* = 369.5 [M+H]^+^; ESI-HRMS: *m/z* [M+H]^+^ calcd for C_17_H_25_N_2_O_5_S: 369.1484; Found: 369.1477.

*(S)-N-(4-(N-(2-Oxotetrahydrofuran-3-yl)*sulfamoyl*)phenyl)benzamide* (**5e**). Compound **5e** was obtained as a white solid (68.1% yield) from compound **4** according to the same procedure used for the synthesis of **5a**. m.p. 222–224 °C; 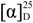 = +9.1° (c = 0.10, CH_3_OH); ^1^H-NMR (DMSO-d_6_): δ 10.62 (1H, s), 8.20 (1H, d, *J* = 8.0 Hz), 8.01–7.95 (4H, m), 7.83–7.81 (2H, d, *J* = 8.8 Hz), 7.57–7.55 (3H, m), 4.38–4.35 (1H, m), 4.23–4.21 (1H, m), 4.12–4.10 (1H, m), 2.14–2.10 (1H, m), 1.86–1.84 (1H, m); ^1^^3^C-NMR (DMSO-d_6_): δ 174.5, 166.7, 142.9, 135.6, 134.5, 131.9, 128.5, 127.8, 127.5, 119.8, 65.1, 51.3, 29.4; EI-MS: *m/z* = 361.4 [M+H]^+^; ESI-HRMS: *m/z* [M+H]^+^ calcd for C_17_H_17_N_2_O_5_S: 361.0858; Found: 361.0855.

*(S)-4-Fluoro-N-(4-(N-(2-oxotetrahydrofuran-3-yl)sulfamoyl)phenyl)benzamide* (**5f**). Compound **5f** was obtained as a white solid (75.6% yield) from compound **4** according to the same procedure used for the synthesis of **5a**. m.p. 236–237 °C; 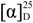 = +10.6° (c = 0.10, CH_3_OH); ^1^H-NMR (DMSO-*d_6_*): δ 10.63 (1H, s), 8.23 (1H, d, *J* = 8.0 Hz), 8.05–7.97 (4H, m), 7.83–7.81 (2H, d, *J* = 8.0 Hz), 7.40 (2H, t, *J* = 9.2 Hz, *J* = 8.4 Hz), 4.38–4.36 (1H, m), 4.23–4.20 (1H, m), 4.12–4.10 (1H, m), 2.14–2.12 (1H, m), 1.85–1.83 (1H, m); ^1^^3^C-NMR (DMSO-*d_6_*): δ 174.4, 165.6, 163.4, 142.6, 135.5, 130.5, 127.4, 119.7, 115.4, 65.0, 51.2, 29.3; EI-MS *m/z* = 379.3 [M+H]^+^; ESI-HRMS: *m/z* [M+H]^+^ calcd for C_17_H_16_FN_2_O_5_S: 379.0764; Found: 379.0764.

*(S)-3-Fluoro-N-(4-(N-(2-oxotetrahydrofuran-3-yl)sulfamoyl)phenyl)benzamide* (**5g**). Compound **5g** was obtained as a white solid (72.4% yield) from compound **4** according to the same procedure used for the synthesis of **5a**. m.p. 201–203 °C; 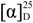 = +9.3° (c = 0.11, CH_3_OH); ^1^H-NMR (DMSO-*d_6_*): δ 10.67 (1H, s), 8.24 (1H, d, *J* = 8.8 Hz), 8.00 (2H, d, *J* = 8.0 Hz), 7.85–7.82 (4H, m), 7.65–7.62 (1H, m), 7.51–7.49 (1H, m), 4.39–4.36 (1H, m), 4.24–4.22 (1H, m), 4.11–4.09 (1H, m), 2.15–2.12 (1H, m), 1.86–1.83 (1H, m); ^1^^3^C-NMR (DMSO-*d_6_*): δ 174.4, 164.6, 163.2, 142.5, 136.7, 135.8, 130.7, 127.5, 123.9, 119.9, 118.7, 114.7, 65.0, 51.2, 29.4; EI-MS: *m/z* = 379.2 [M+H]^+^; ESI-HRMS: *m/z* [M+H]^+^ calcd for C_17_H_16_FN_2_O_5_S: 379.0764; Found: 379.0763.

*(S)-2-Fluoro-N-(4-(N-(2-oxotetrahydrofuran-3-yl)sulfamoyl)phenyl)benzamide* (**5h**). Compound **5h** was obtained as a white solid (70.7% yield) from compound **4** according to the same procedure used for the synthesis of **5a**. m.p. 205–207 °C; 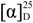 = +11.7° (c *=* 0.12, CH_3_OH) for 99.2% e.e. purity material. HPLC (chiral sample) DAICEL AYH Chiralpak, 70:30 hexane/ EtOH, flow rate of 1 mL/min; retention time of the major enantiomer 41.545 min, minor enantiomer 25.533 min; HPLC (Racemic sample) DAICEL AYH Chiralpak, 70:30 hexane/EtOH, flow rate of 1 mL/min; retention times of 25.475 and 41.422 min; ^1^H-NMR (DMSO-*d_6_*): δ 10.84 (1H, s), 8.24 (1H, d, *J* = 8.4 Hz), 7.93 (2H, d, *J* = 8.0 Hz), 7.83 (2H, d, *J* = 8.8 Hz), 7.71–7.69 (1H, m), 7.60–7.59 (1H, m), 7.37–7.35 (2H, m) 4.38–4.35 (1H, m), 4.23–4.21 (1H, m), 4.11–4.09 (1H, m), 2.14–2.12 (1H, m), 1.83–1.82 (1H, m); ^1^^3^C-NMR (DMSO-*d_6_*): δ 174.6, 163.4, 160.1, 142.7, 135.9, 133.0, 130.0, 128.5, 127.7, 124.7, 124.5, 119.4, 116.4, 65.2, 51.4, 29.5; EI-MS: *m/z* = 379.4 [M+H]^+^; ESI-HRMS: *m/z* [M+H]^+^ calcd for C_17_H_16_FN_2_O_5_S: 379.0764; Found: 379.0762.

*(S)-4-chloro-N-(4-(N-(2-oxotetrahydrofuran-3-yl)sulfamoyl)phenyl)benzamide* (**5i**). Compound **5i** was obtained as a white solid (68.5% yield) from compound **4** according to the same procedure used for the synthesis of **5a**. m.p. 256–258 °C; 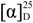 = +11.3° (c = 0.11, CH_3_OH); ^1^H-NMR (DMSO-*d_6_*): δ 10.67 (1H, s), 8.20 (1H, d, *J* = 8.0 Hz), 8.01–7.98 (4H, m), 7.84 (4H, d, *J* = 9.2 Hz), 7.65 (2H, d, *J* = 8.4 Hz), 4.39–4.36 (1H, m), 4.23–4.21 (1H, m), 4.11–4.09 (1H, m), 2.14–2.13 (1H, m), 1.86–1.83 (1H, m); ^1^^3^C-NMR (DMSO-d_6_): δ 175.1, 165.5, 143.2, 137.4, 136.3, 133.7, 130.3, 129.1, 128.1, 120.4, 65.1, 51.8, 30.0; EI-MS: *m/z* = 395.2 [M+H]^+^; ESI-HRMS: *m/z* [M+H]^+^ calcd for C_17_H_16_ClN_2_O_5_S: 395.0468; Found: 395.0474.

*(S)-3-Chloro-N-(4-(N-(2-oxotetrahydrofuran-3-yl)sulfamoyl)phenyl)benzamide* (**5j**). Compound **5j** was obtained as a white solid (71.2% yield) from compound **4** according to the same procedure used for the synthesis of **5a**. m.p. 213–214 °C; 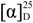 = +10.4° (c = 0.10, CH_3_OH); ^1^H-NMR (DMSO-*d_6_*): δ 10.74 (1H, s), 8.25 (1H, d, *J* = 8.4 Hz), 8.02–8.00 (6H, m), 7.91–7.90 (1H, m), 7.85–7.82 (1H, m), 7.72–7.69 (1H, m), 7.62–7.58 (1H, m), 4.40–4.38 (1H, m), 4.24–4.22 (1H, m), 4.12–4.11 (1H, m), 2.15–2.14 (1H, m), 1.86–1.83 (1H, m); ^1^^3^C-NMR (DMSO-d_6_): δ 174.6, 164.7, 142.6, 136.4, 135.9, 133.3, 131.8, 130.6, 127.6, 126.7, 119.9, 65.2, 51.4, 29.5; EI-MS: *m/z* = 395.3 [M+H]^+^; ESI-HRMS: *m/z* [M+H]^+^ calcd for C_17_H_16_ClN_2_O_5_S: 395.0468; Found: 395.0468.

*(S)-2-Chloro-N-(4-(N-(2-oxotetrahydrofuran-3-yl)sulfamoyl)phenyl)benzamide* (**5k**). Compound **5k** was obtained as a white solid (69.2% yield) from compound **4** according to the same procedure used for the synthesis of **5a**. m.p. 204–205 °C; 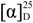 = +12.5° (c = 0.12, CH_3_OH); ^1^H-NMR (DMSO-*d_6_*) δ ppm: 10.93 (1H, s), 8.25 (1H, d, *J* = 8.0 Hz), 7.92 (2H, d, *J* = 8.0 Hz), 7.84 (2H, d, *J* = 8.0 Hz), 7.59–7.50 (4H, m), 4.38–4.36 (1H, m), 4.24–4.20 (1H, m), 4.11–4.09 (1H, m), 2.51–2.50 (1H, m), 1.87–1.84 (1H, m); ^1^^3^C-NMR (DMSO-*d_6_*) δ: 175.0, 166.0, 142.9, 137.0, 136.4, 132.0, 130.4, 130.3, 129.5, 128.3, 127.9, 119.8, 65.7, 51.9, 30.0; EI-MS: *m/z* = 395.2 [M+H]^+^; ESI-HRMS: *m/z* [M+H]^+^ calcd for C_17_H_16_ClN_2_O_5_S: 395.0468; Found: 395.0464.

*(S)-N-(4-(N-(2-Oxotetrahydrofuran-3-yl)sulfamoyl)phenyl)-2-(trifluoromethyl)benzamide* (**5l**). Compound **5l** was obtained as a white solid (68.0% yield) from compound **4** according to the same procedure used for the synthesis of **5a**. m.p. 186–187 °C; 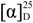 = +11.2° (c = 0.10, CH_3_OH); ^1^H-NMR (DMSO-*d_6_*): δ 10.99 (1H, s), 8.26 (1H, d, *J* = 8.0 Hz), 7.88–7.82 (6H, m), 7.77–7.70 (2H, m), 4.39–4.36 (1H, m), 4.24–4.22 (1H, m), 4.11–4.09 (1H, m), 2.17–2.15 (1H, m), 1.88–1.83 (1H, m); ^1^^3^C-NMR (DMSO-*d_6_*) δ: 174.6, 166.1, 142.4, 136.0, 136.2, 132.8, 130.4, 128.6, 127.7, 126.5, 126.0, 125.1, 122.4, 119.3, 65.2, 51.3, 29.5; EI-MS: *m/z* = 429.4 [M+H]^+^; ESI-HRMS: *m/z* [M+H]^+^ calcd for C_18_H_16_F_3_N_2_O_5_S: 429.0732; Found: 429.0724.

### 3.3. Evaluation of the Biological Activity

For the primary screening of the synthesized compounds, the inhibitory activities of the compounds towards violacein production were determined according to a spot test method using C_10_-HSL as the positive control. Briefly, CV026 (400 μL) were cultured in LB medium. The PBS solution of C_6_-HSL (15 μL, 0.125 mM) was then added and the resulting mixture was gently mixed with molten semi-solid LB agar (5 mL). The mixture was then overlaid on the surface of the solid LB agar. Upon solidification of the top layer, the test compounds were spotted on the plate. The plates were then incubated overnight at 30 °C before being cultured for 16–18 h. The activities of the compounds inhibiting violacein production were detected by the presence of white colonies/holes in a purple background of the plates [12,22]. If the growth inhibition zones were observed, it would imply that the compounds had the growth-inhibitory effect.

For the secondary screening of the compounds which inhibited violacein production, CV026 were cultured in LB medium and subsequently placed in 12-well plates together with the LB medium. The PBS solution of C_6_-HSL (15 μL, 0.125 mM) and different concentrations of the test compounds were then added to the wells. Following an incubation period of 16–18 h, a portion (1 mL) of the culture medium was removed from each mixture and centrifuged at 12,470 × *g* for 10 min to precipitate the insoluble violacein. The supernatant from the culture was then discarded and DMSO (500 μL) was added to each Eppendorf tube to completely solubilize the violacein. The resulting mixtures were then centrifuged at 12,470 × *g* for 10 min to remove the cells. A sample (200 μL) of the upper layer of the violacein solution was carefully removed and placed into a 96-well microplate, and its absorbance was measured at 585 nm [23]. The results expressed as IC_50_ (half maximum inhibitory concentration) were taken to be the average of three determinations and were calculated using the Origin 8.0 software.

## 4. Conclusions

In conclusion, we have developed a novel series of *N*-sulfonyl homoserine lactone derivatives **5a**–**l** and evaluated their QS inhibitory activities towards violacein production. The enantiopurity of compound **5h** in particular (99.2% e.e.) demonstrated that compounds with a high level of enantiomeric purity could be synthesized according to this method. Preliminary SAR studies showed that compounds bearing substituted aryl groups at the R position displayed better levels of inhibitory activity than those bearing alkyl groups at the same position. Of the compounds tested, compounds **5h** and **5k** bearing *ortho*-halogen-substituted aryl groups at the R position exhibited promising levels of inhibitory activity against violacein production and are currently being considered as promising lead compounds for further structural modification studies.

## Supplementary Materials

Supplementary materials can be accessed at: http://www.mdpi.com/1420-3049/18/3/3266/s1.
