# Supplementary material for: Design, Synthesis and Biological Evaluation of N-Sulfonyl Homoserine Lactone Derivatives as Inhibitors of Quorum Sensing in Chromobacterium violaceum
Source: Molecules. 2013 Mar 13;18(3):3266–78. doi: 10.3390/molecules18033266 (PMC6270181; doi:10.3390/molecules18033266)

Supplementary Materials

Figure S1. The ^{1}H-NMR spectrum of **5a**.

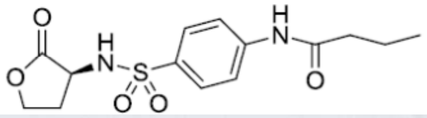

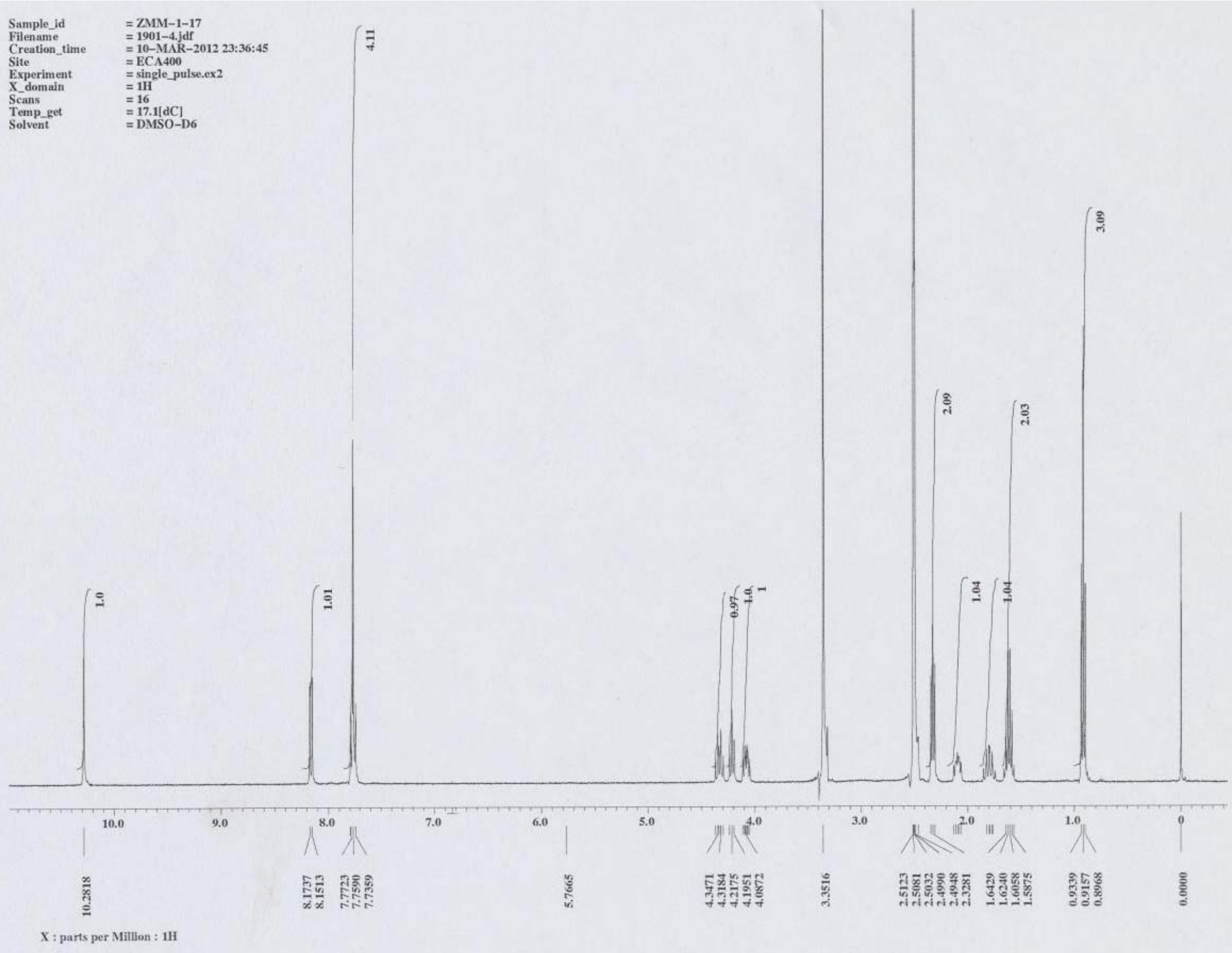

Figure S2. The ^{1}H-NMR spectrum of **5b**.

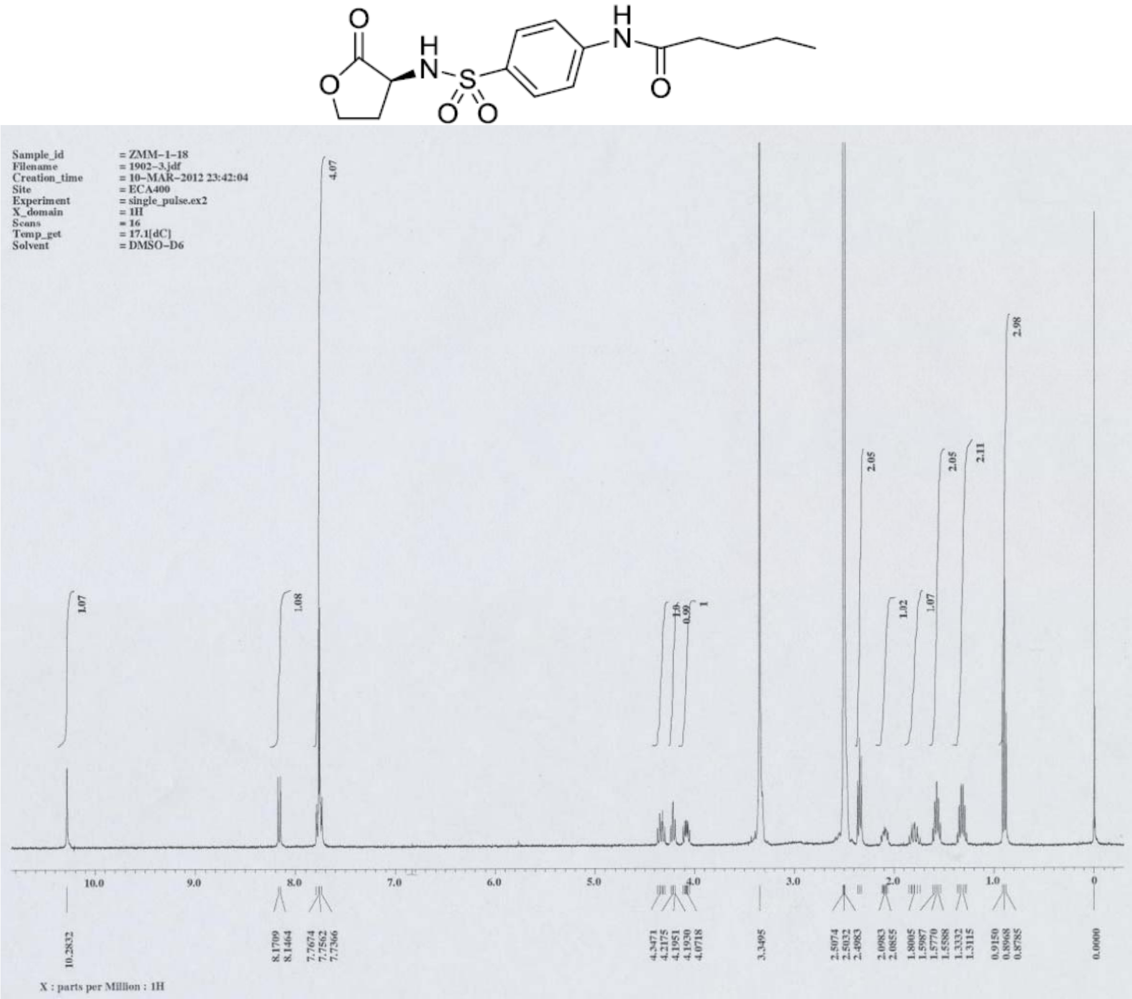

Figure S3. The ^{1}H-NMR spectrum of **5c**.

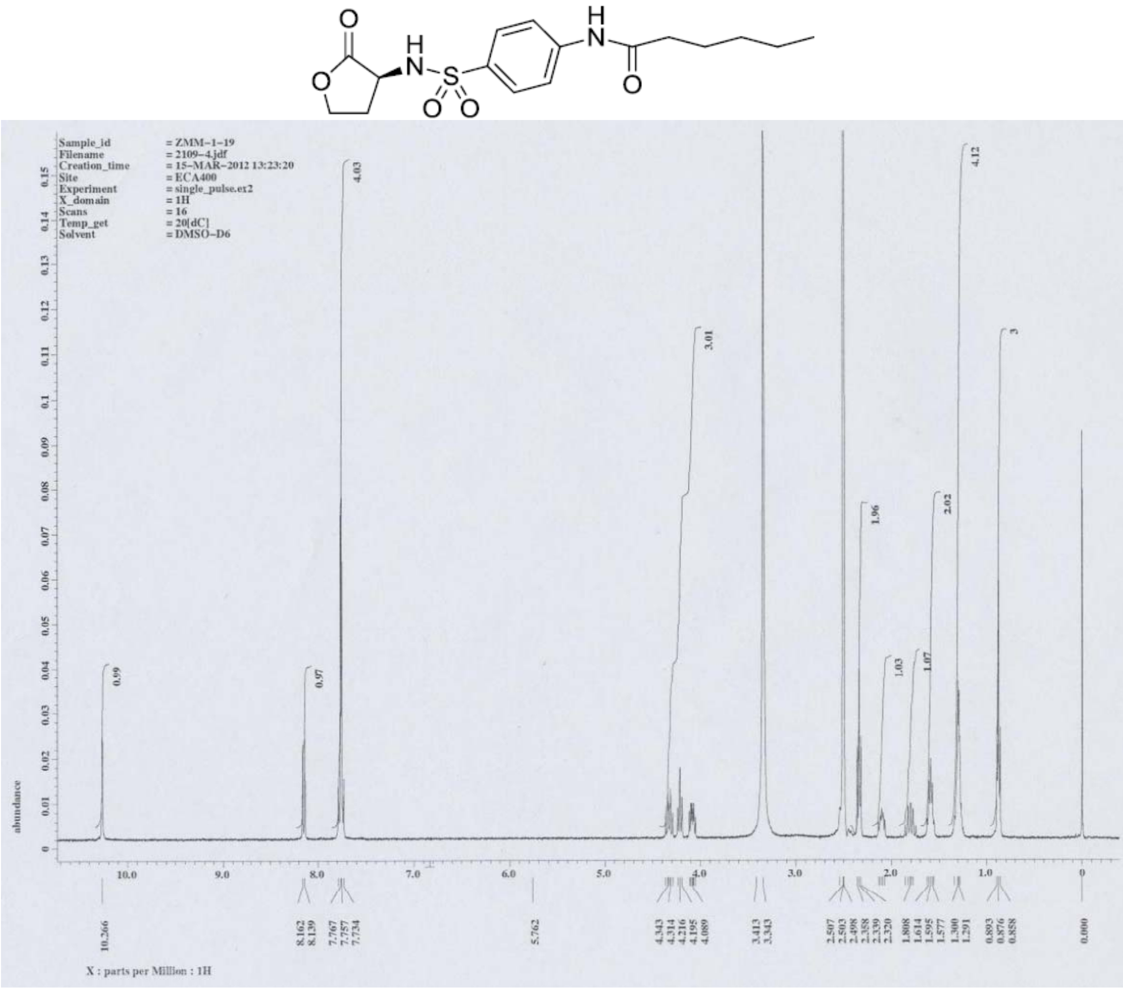

Figure S4. The ^{1}H-NMR spectrum of **5d**.

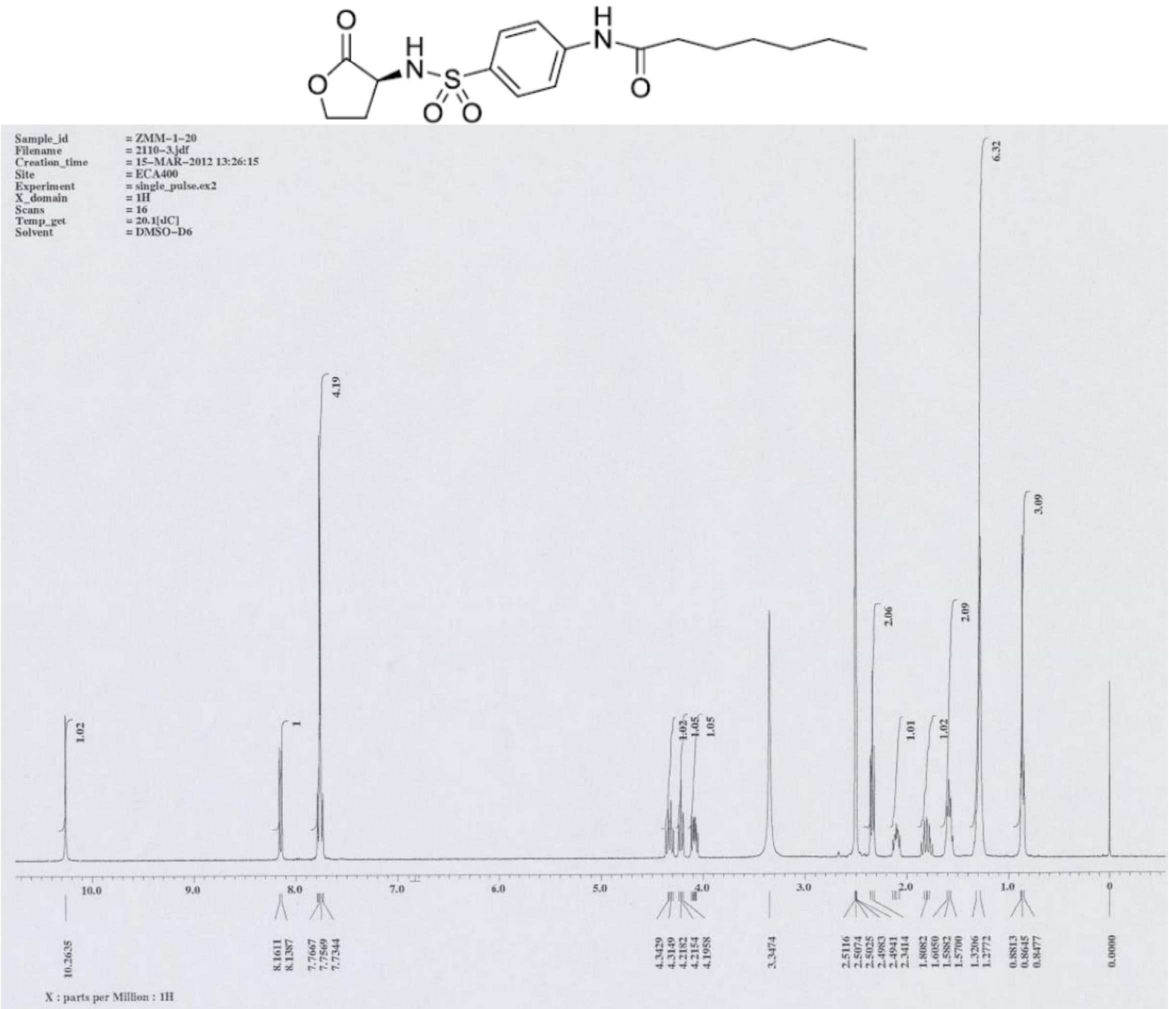

Figure S5. The ^{1}H-NMR spectrum of **5e.**

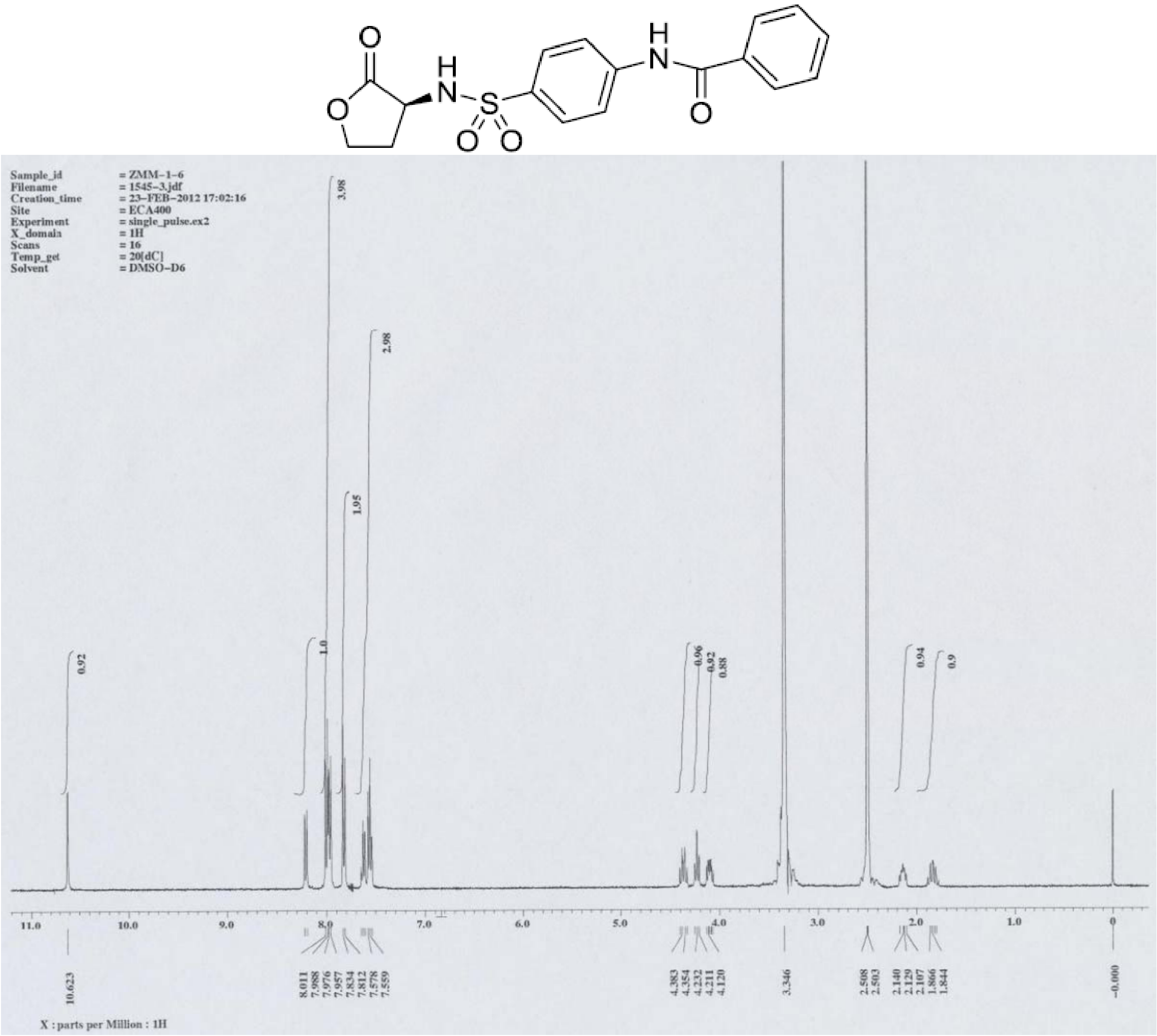

Figure S6. The ^{1}H-NMR spectrum of **5f**.

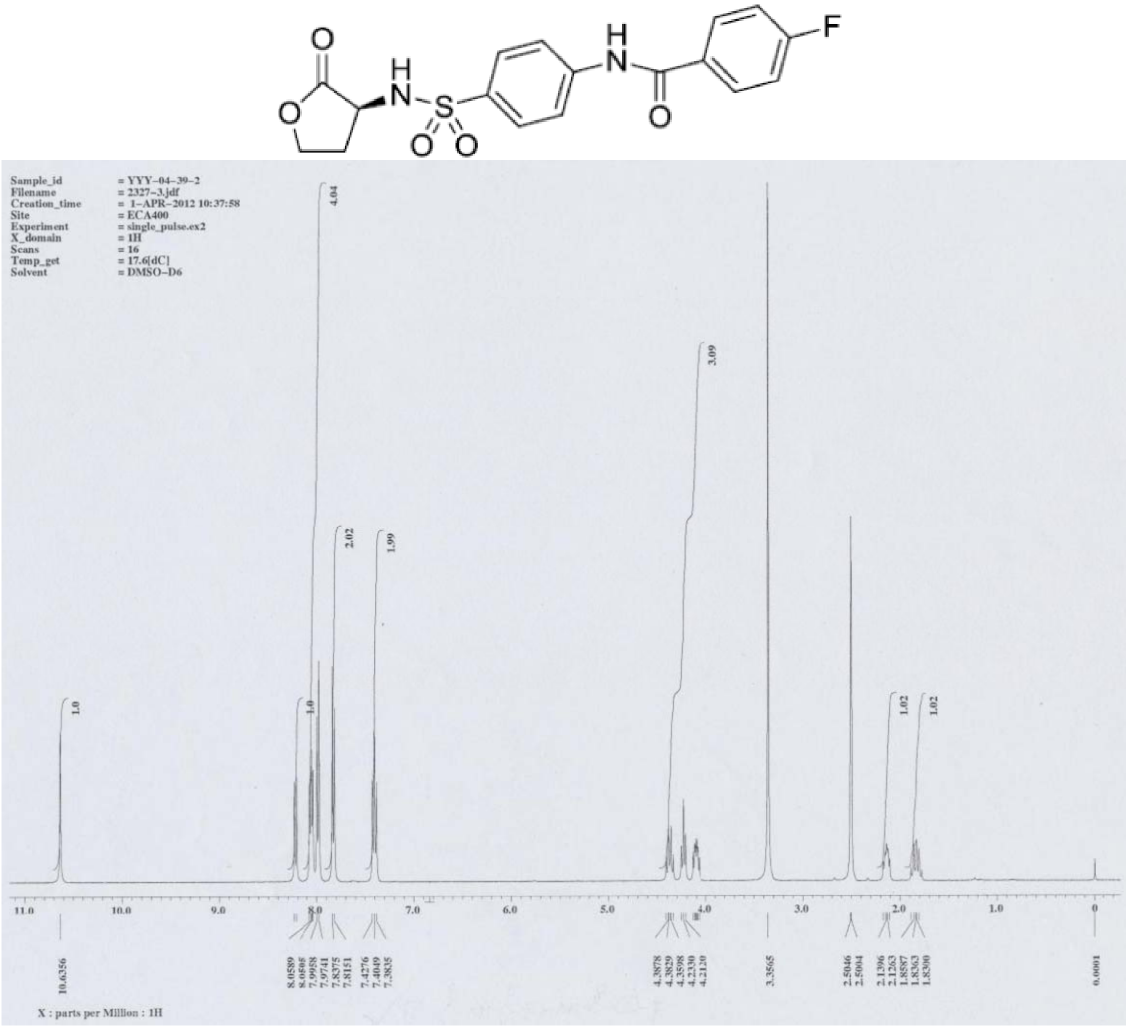

Figure S7. The ^{1}H-NMR spectrum of **5g.**

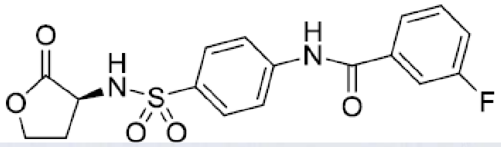

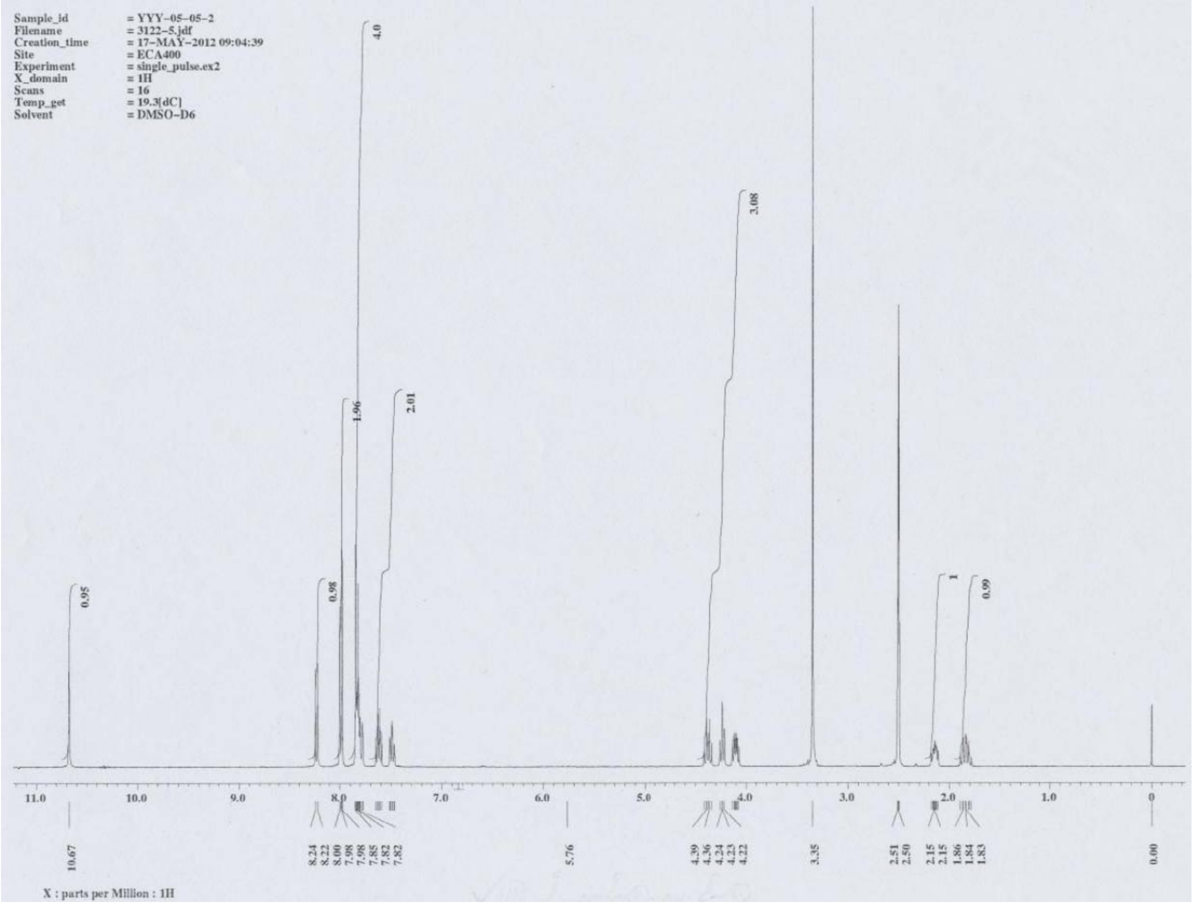

Figure S8. The ^{1}H-NMR spectrum of **5h**.

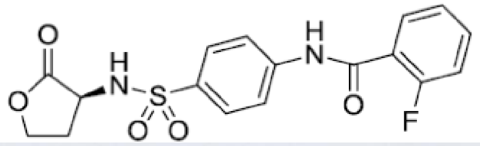

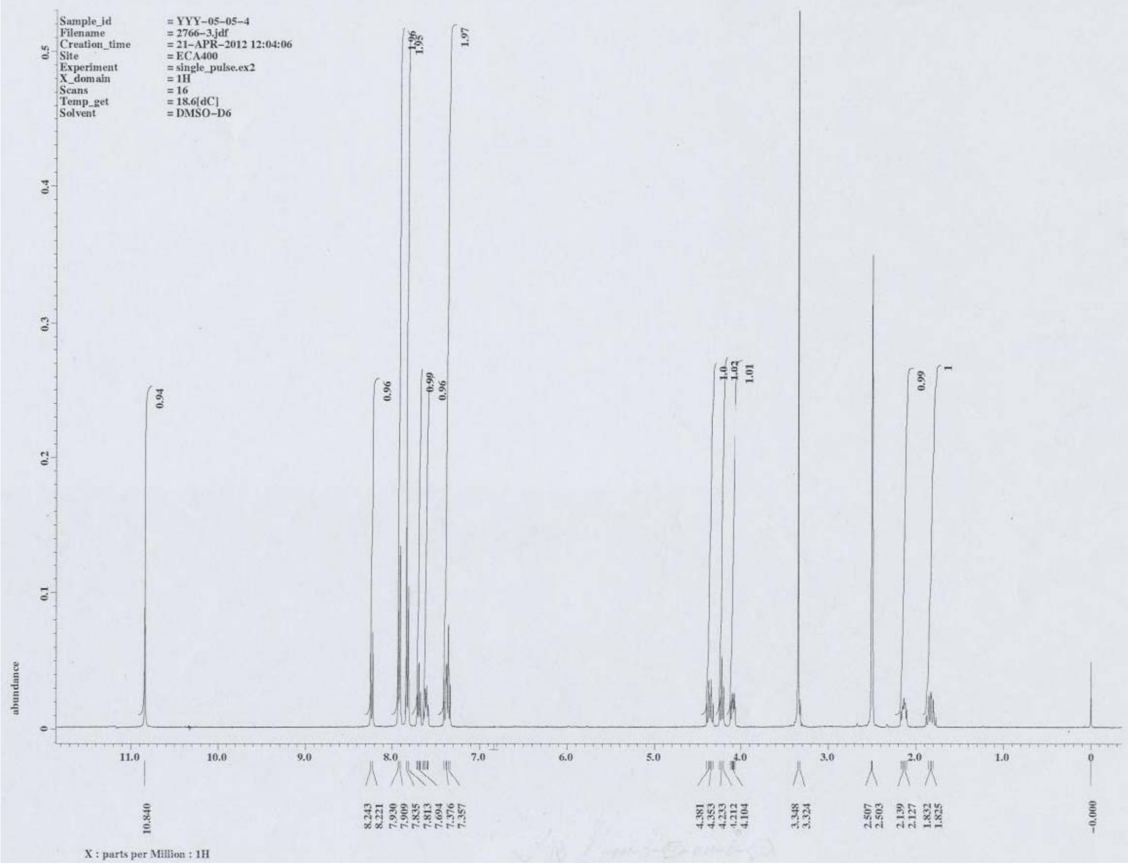

Figure S9. The ^{1}H-NMR spectrum of **5i**.

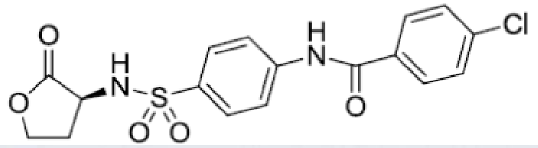

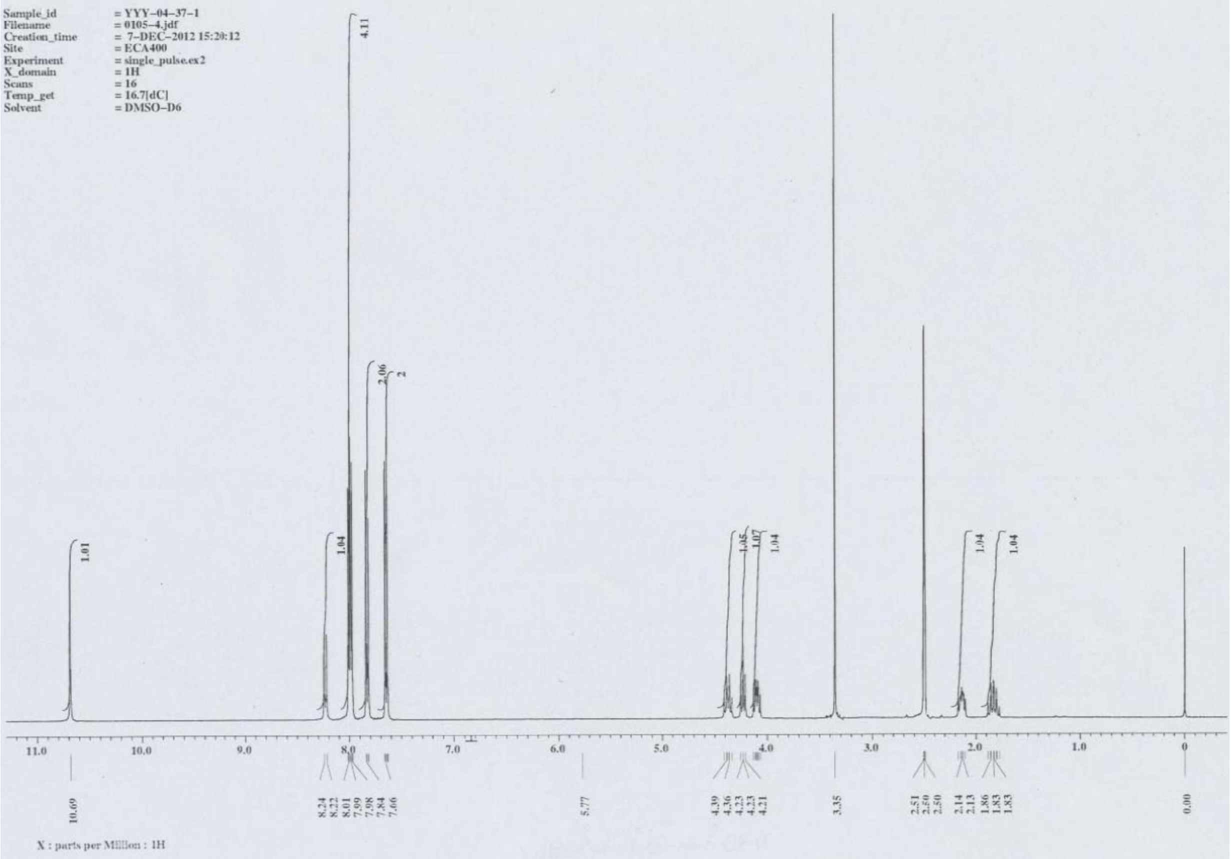

Figure S10. The ^{1}H-NMR spectrum of **5j**.

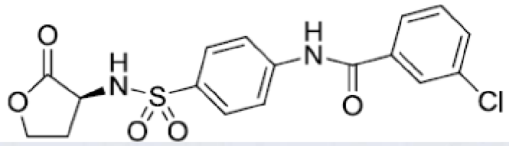

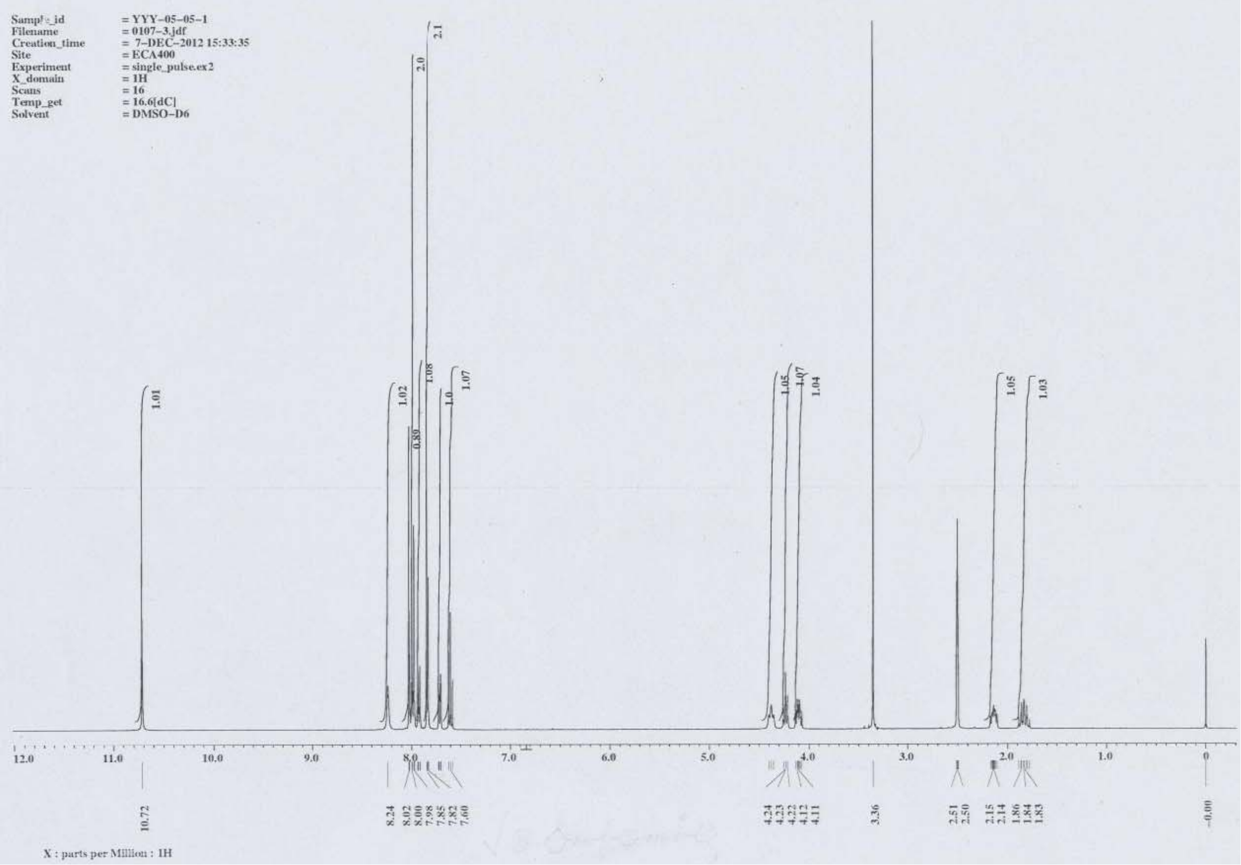

Figure S11. The ^{1}H-NMR spectrum of **5k**.

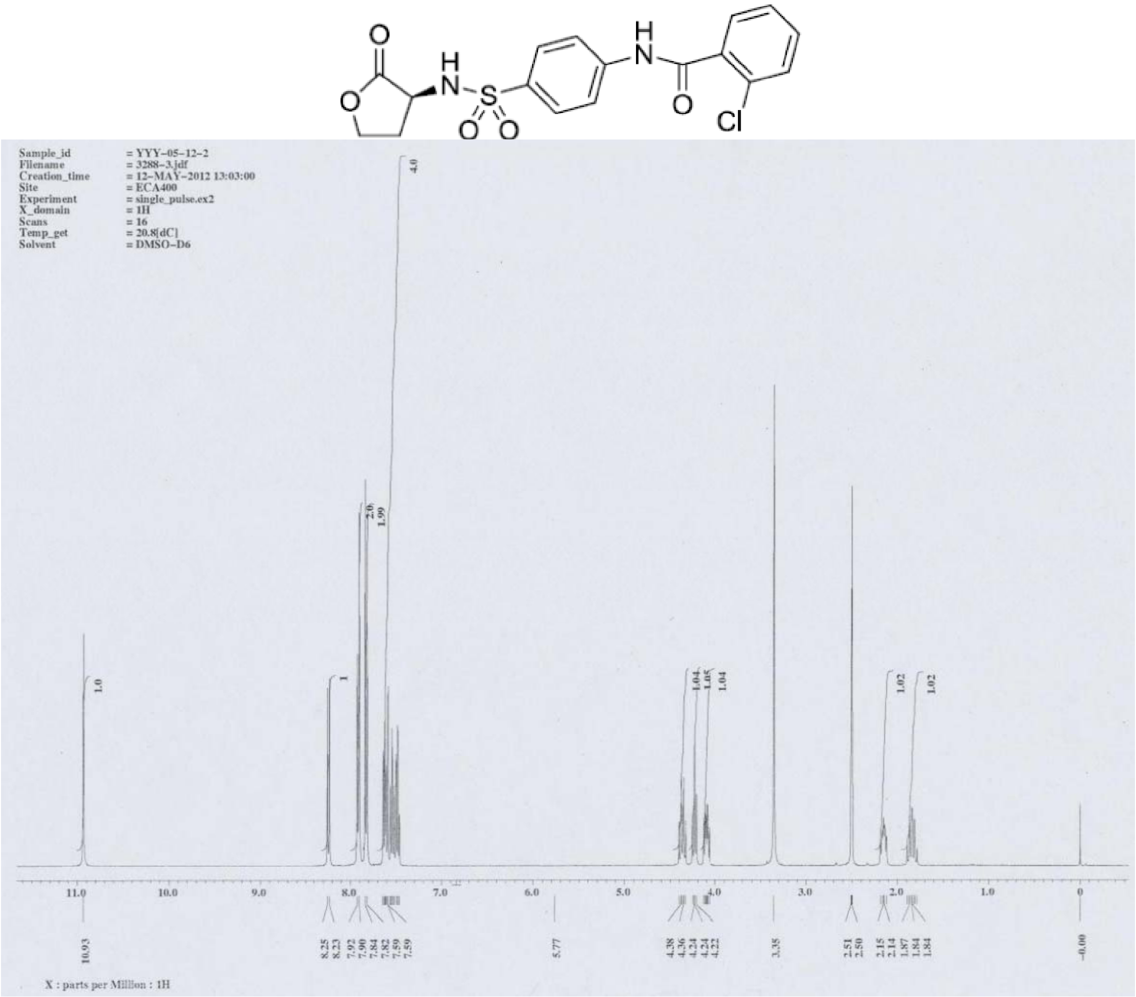

Figure S12. The ^{1}H-NMR spectrum of **5l**.

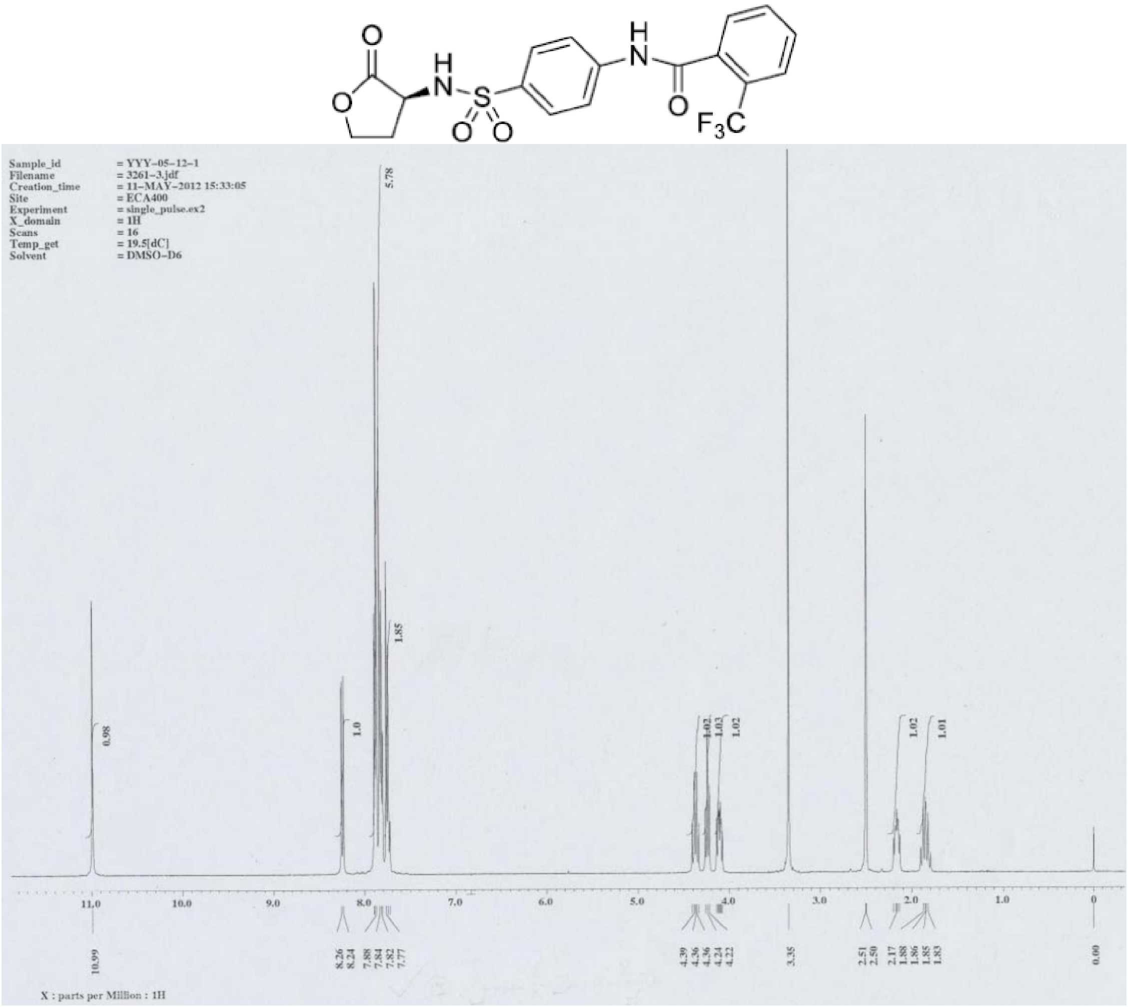

Supplement: Supplementary file 1 [file molecules-18-03266-s001.pdf]
